# Closing Developmental Gaps: Effectiveness of Community-Based Early Intervention for Young Children with Developmental Delays

**DOI:** 10.3390/children13040459

**Published:** 2026-03-27

**Authors:** Melissa Gonzalez, Morgan D. Darabi, Paris Rayneri, Elana Mansoor, Rachel Spector, Ruby Natale

**Affiliations:** 1 Department of Pediatrics, University of Miami Miller School of Medicine, Miami, FL 33136, USA; mdarabi@miami.edu (M.D.D.); parisrayneri@miami.edu (P.R.); emansoor@med.miami.edu (E.M.); rnatale@med.miami.edu (R.N.); 2 The Children’s Trust, Miami, FL 33129, USA; rachel@thechildrenstrust.org

**Keywords:** early intervention, mild developmental delays, community-based programs, developmental outcomes, underserved populations, preschool-aged children

## Abstract

**Highlights:**

**What are the main findings?**

- Eighty-five percent (85%) of young children with mild developmental delays met program-defined criteria for improvement following short-term, community-based intervention.
- Comparable rates of improvement were observed across service types and most family characteristics.

**What are the implications of the main findings?**

- Community-funded programs may help address services gaps for children ineligible for IDEA Part C or Part B funded services.
- Short-term early intervention services may contribute to equity in access to developmental support.

**Abstract:**

Background/Objectives: Early intervention is associated with improved outcomes for young children with developmental delays, yet many with mild delays are ineligible for services under the Individuals with Disabilities Education Act (IDEA). The Early Discovery (ED) Program addressed this gap by providing short-term, targeted intervention for children ages 0–5 who did not qualify for publicly funded services. This study evaluated program outcomes across intervention types. Methods: During 2024–2025, 342 families completed the ED Program, receiving one of the following: speech-language (68%), general developmental (12%), occupational (14%), or behavioral (6%) intervention across 8–20 sessions. Eligibility required Miami-Dade residency and ineligibility for IDEA-funded services. Standardized pre- and post-intervention assessments were analyzed using descriptive statistics, correlations, and group comparisons. Results: Most households reported incomes <$70,000 (71%), with many experiencing additional risk factors including prematurity (15%), public or no insurance (47%), limited English proficiency (21%), and single-caregiver households (30%). Overall, 85% of children met criteria for improvement. Improvement rates varied by child ethnicity. No statistically significant differences were observed by child age, race, gender, prematurity, insurance status, caregiver demographics, household characteristics, or intervention type. Sensitivity analyses largely confirmed the primary findings, with ethnicity no longer significant and younger age emerging as a significant predictor of improvement. Conclusions: Findings suggest short-term, targeted intervention may support developmental progress among young children with mild delays who would otherwise remain unserved. Community-based programs such as ED may play a critical role in advancing developmental equity by reaching children with developmental and socioeconomic risk factors prior to school entry.

## 1. Introduction

Early childhood is a critical developmental period marked by rapid brain growth that sets the foundation for cognitive development [1]. During these years, heightened neuroplasticity makes early interventions particularly beneficial. Research consistently demonstrates early intervention services are associated with improved developmental outcomes across multiple domains [2,3]. On average, children who received evidence-based support displayed better cognitive, language, and motor development than their peers [3] as well as enhanced social-emotional skills and greater readiness for school entry [2]. These findings underscore early childhood as a uniquely responsive period in which timely intervention can meaningfully shape developmental trajectories.

However, some researchers argue that developmental gains observed in young children may be the result of natural maturation rather than intervention effects, given the rapid developmental changes that occur during early childhood [4]. This notion challenges the necessity of short-term intervention and highlights the need to distinguish intervention-related change from typical maturation. A Response to Intervention (RTI) framework, conceptualizes early intervention as a needs-based approach in which targeted, brief supports are matched to a child’s level of functioning and adjusted based on progress. Intervention intensity and strategies are individualized and responsive to developmental needs. Consequently, RTI-informed models are designed to promote meaningful gains across diverse service types, even within relatively short intervention periods [5].

Early intervention has also been linked to lasting benefits beyond early childhood [6]. Programs focused on early learning and self-regulation have been associated with higher academic achievement, improved mental health and greater school success, along with fewer behavioral difficulties during adolescence [7]. Early support has also been linked to lower rates of involvement in the criminal justice system, while preschool suspension and expulsion are associated with an increased risk of subsequent involvement in the juvenile justice system [8]. Importantly, delays in access to early intervention increase the likelihood that developmental challenges will persist and exacerbate over time, emphasizing the societal and individual costs of missed opportunities for early support [9].

Despite strong evidence demonstrating the benefits of early intervention, access to these services remains inconsistent. Children with mild to moderate developmental delays are particularly likely to be overlooked and are significantly less likely to receive early intervention services than children with more severe impairments [10]. As a result, many at-risk children miss critical support during formative developmental periods. Addressing these gaps is essential, as even mild delays can compound over time and negatively affect multiple developmental domains if left unaddressed.

### 1.1. Limitations in Current Systems

Eligibility for federally funded early intervention under Individuals with Disabilities Education Act (IDEA) Parts C and B is determined at the state level, resulting in variability in criteria and the frequent exclusion of children with mild developmental delays. Consequently, many children who could benefit from early support do not meet formal eligibility criteria and receive little to no intervention [10]. These gaps disproportionately affect children from low-income and historically marginalized communities, where stricter eligibility requirements intersect with additional barriers to care [11,12]. To examine how these structural factors may shape access and outcomes, the present study was conceptually informed by the Equity Action Framework, which emphasizes examining structural barriers, resource distribution, and differential impacts to evaluate equity within early childhood systems [13].

Socioeconomic factors such as limited insurance coverage, financial strain, and reduced access to supportive services further intensify these disparities, placing already vulnerable children at an even greater risk for unmet developmental needs. These system-level barriers highlight the importance of community-based approaches that provide timely, preventive supports for children with mild delays who do not qualify for IDEA services but may benefit from early, targeted intervention [10,14].

### 1.2. Community-Based Programs

Local and community-funded programs play an important role in access to much- needed services for children with developmental delays. These programs tend to be more flexible in eligibility and are often embedded within community settings [15]. Community-funded programs are also better able to respond quickly to developmental concerns, reducing wait times and providing early and preventative services that can mitigate delays prior to kindergarten enrollment [16]. However, programs specifically designed for children with mild developmental delays remain limited.

Longitudinal studies examining brief community-based interventions for children at-risk for developmental delays, show that early gains in self-regulation and preacademic skills are associated with sustained academic and behavioral benefits [17]. Existing community-based intervention studies have primarily focused on children with identified medical risk or diagnosed developmental delays. Home-based early developmental interventions have demonstrated cognitive gains among at-risk infants [18], routines-based models have improved functional outcomes for children with or at-risk for delay [19], and center-based programs have shown gains in language and behavior among socially disadvantaged families [20]. Other studies demonstrate that short-term, parent-implemented early interventions can produce significant improvements in receptive language outcomes for toddlers with language delays, even in the absence of a formal diagnosis [21].

While these findings support community-based intervention, prior studies typically target specific risk groups or single developmental domains. Few studies have examined brief, community-based models spanning multiple service types for children with mild delays who do not qualify for IDEA-funded services. Similar gap-filling initiatives exist that provide short-term, community-based intervention for young children with undiagnosed or mild developmental delays who do not meet IDEA eligibility criteria. These programs emphasize parent coaching, individualized goal setting, and school-readiness outcomes [22]. However, while these initiatives are similar, few have published outcome data evaluating the success of brief intervention models specifically for child with mild delays who do not qualify for IDEA-funded services. This highlights the importance of the Early Discovery program in addressing a critical gap for families who need support but do not qualify for intensive services [23].

### 1.3. The Early Discovery Program

The Early Discovery (ED) Program is a short-term, community-based early intervention program serving children from birth to five years of age with mild developmental delays. Funded by The Children’s Trust, a nonprofit organization that partners with local agencies to support children and families in Miami-Dade County, the program was specifically designed to address gaps in access to early intervention services for children who do not qualify for IDEA Part C or Part B. ED prioritizes children who might otherwise remain unserved, and provides individualized general developmental, speech-language, behavioral, or occupational intervention services to address early concerns and promote positive developmental outcomes. Services are delivered through community-based providers to maximize accessibility for children and their families across the county.

The ED Program is conceptually grounded in a Response to Intervention (RTI) framework, which provides a theoretical rationale for brief, targeted intervention [24]. RTI emphasizes early identification of developmental concerns, individualized service delivery, and data-informed decision-making to address emerging difficulties before they become more persistent or severe [5]. Within this framework, children’s response to intervention informs the level and duration of support, with the goal of strengthening foundational skills during periods of rapid neurodevelopment and by preventing the progression of delays [25]. Developmental domains are dynamic and interconnected. Therefore, improvements in core areas such as communication, attention, or self-regulation may generalize to broader functioning across service types [26].

Unlike many community-based early intervention programs that provide fixed-duration services or focus primarily on parent education, the ED Program uses an individualized service model guided by children’s response to intervention. Services vary in intensity and duration based on ongoing progress monitoring, allowing intervention to be adjusted to child need. This RTI-informed structure emphasizes early, preventive support and data-informed decision-making to address emerging developmental concerns before they require more intensive services.

### 1.4. Purpose

The purpose of this study was to examine observed developmental change associated with participation in the ED Program, a community-funded early intervention initiative serving young children from low-income and historically marginalized communities with mild developmental delays who do not qualify for IDEA Part C or Part B. Effectiveness was defined as statistically significant pre- to post-intervention improvement in children’s developmental functioning as measured by standardized developmental assessments. Despite the well-established benefits of early intervention, limited evidence exists regarding the impact of short-term programs for children ineligible for federally funded services. Children enrolled in the ED Program received 8–20 sessions of speech-language, occupational, behavioral, or general developmental intervention. This study examined developmental outcomes across service types and explored associations between improvement and child and family characteristics, including age, gender, socioeconomic risk factors, and intervention type. By evaluating children from financially constrained families, the study also explored whether developmental gains were distributed comparably across groups historically affected by disparities in early intervention access.

We hypothesized most children enrolled in the ED Program would demonstrate significant pre- to post-test improvement, with no significant differences in outcomes across service types. The ED Program uses an RTI-informed, individualized service model in which intervention is tailored to children’s needs and progress, supporting comparable developmental gains across service types. In addition, the program’s community-based design and efforts to reduce structural barriers to access may promote equitable outcomes across diverse demographic and socioeconomic groups. Consistent with this framework, we further hypothesized that the program would support developmental gains equitably across a diverse population, with no significant differences in improvement by child or family characteristics. This expectation reflects the program’s RTI-informed, individualized service model, in which intervention is tailored to children’s needs and progress, supporting comparable gains across service types. Lastly, we hypothesized that number of sessions would not significantly predict improvement outcomes.

## 2. Materials and Methods

### 2.1. Program Design and Setting

The ED Program is funded through a local children’s services organization, The Children’s Trust, and operates in partnership with community agencies to serve young children with mild delays who are ineligible for IDEA Part C or Part B services. Data were collected as part of routine program implementation. As this was not funded as a research program but rather a community service grant. This study used a single group pre-post program evaluation design without a comparison group. The purpose was to examine developmental outcomes for children enrolled in the ED Program, a community-based early intervention initiative in Miami-Dade County. Standardized assessments were administered at intake and at program discharge to evaluate change over time. All standardized assessments used age-normed scoring (e.g., standard scores, T-scores) based on age-specific normative samples. These scores adjusted for expected developmental maturation, so changes reflected performance relative to same-age peers.

### 2.2. Program Services

Speech-Language. Speech and language intervention services are provided for children who primarily demonstrate difficulties with expressive and/or receptive language, as determined by below average scaled scores on the Preschool Language Scale–5th Edition (PLS–5; Pearson, San Antonio, TX, USA). Licensed speech-language pathologists or supervised speech-language pathology assistants deliver services in the child’s natural environment or clinic-based settings, depending on family preference and child needs. Intervention is based on the Hanen method of speech services, which is an evidence-based treatment approach that focuses on responsive caregiver-child interactions [27]. Hanen strategies support language development by embedding intervention within daily routines and play, using techniques such as following the child’s lead, commenting rather than questioning, interpreting and expanding child communication, and modeling language [28]. This approach has been shown to improve children’s expressive and receptive language, social communication, and caregiver responsiveness [28,29].

Occupational. Occupational intervention services are available for children who demonstrate challenges in fine and gross motor skills, sensory processing, or functional use of their hands (e.g., grasping objects, manipulating tools), as determined by below average scores on the Peabody Developmental Motor Scales, Second Edition (PDMS-2; PRO-ED, Austin, TX, USA). Occupational intervention practitioners collaborate with caregivers to support independence and skill development in daily activities. Services are delivered in home, childcare, or clinic-based settings and are guided by the Occupational Therapy Practice Framework: Domain and Process (4th ed.) [30]. This evidence-based framework emphasizes client-centered, goal-directed intervention to promote participation, functional performance, and engagement across everyday routines and environments.

General Developmental. General developmental intervention services are provided to children whose developmental needs are not more appropriately addressed within a single, discipline-specific service type (e.g., speech-language, occupational, or behavioral intervention), although some overlap in intervention strategies may occur. These services support children with mild delays across one or more developmental domains, including motor, cognitive or academic, language, social-emotional, and daily living skills as determined by below average standard scores on the Brigance Inventory of Early Development (Curriculum Associates, North Billerica, MA, USA). Qualified developmental interventionists deliver individualized, developmentally informed support to promote overall developmental progress and functional skill acquisition in naturalistic settings (e.g., childcare centers or homes). Services are based on best practices that align with the Early Start Denver Model (ESDM) of developmental intervention [31]. The ESDM is an evidence-based, play-based early intervention approach integrating developmental science and applied behavior analysis principles. ESDM emphasizes relationship-based, data-driven strategies embedded within daily routines to promote social-emotional, language, and cognitive development in young children with developmental delays [31].

Behavioral. Behavioral intervention services support children who experience challenges related to social-emotional development, emotional regulation, or peer interactions as determined by elevated T-scores on the Devereux Early Childhood Assessment (DECA; Kaplan Early Learning Company, Lewisville, NC, USA). Behavioral specialists work collaboratively with children and caregivers to strengthen protective factors, reduce behavioral concerns, and promote adaptive social-emotional skills. Services are provided in home or childcare settings and are informed by evidence-based behavioral interventions, including Parent-Child Interaction Therapy (PCIT) and Parent Management Training (PMT) [32,33]. These approaches focus on coaching caregivers in effective interaction, positive behavior support, and consistent behavior management strategies to improve child emotional regulation, social competence, and caregiver-child relationships.

### 2.3. Participants

During the 2024–2025 fiscal year, 342 children (33% female, M_age_ = 28 months at intake) and their caregivers (M_age_ = 32 years old at child’s birth) completed the ED Program. See Table 1 for complete demographic information. This study was reviewed by the University of Miami Human Subject Research Office and determined to be exempt from full review (Approval # 20250933), as it involved program evaluation using de-identified data. To qualify for the program, children must be 0 to 5 years old, reside in Miami-Dade County, and be deemed ineligible for Part C or Part B services defined by the IDEA. Additionally, children were required to fall within the below average range on a pre-intervention assessment (i.e., standard scores between 71 and 84 or a 10–29% developmental delay). Children with diagnosed disorders known to impact development (e.g., hearing loss, autism spectrum disorder, cerebral palsy) were excluded from the study as they were eligible to receive Part C or Part B services and this program was intentionally developed to fill a service gap for children at-risk for developmental delays. Children were referred to the ED Program through multiple sources. The majority of referrals to the ED Program are from local Part C and Part B early intervention programs, Early Steps North, Early Steps South, and Miami Dade County Public School Systems, following determination of ineligibility for federally funded services. Other referrals were received from local community agencies, childcare providers, and self-referrals by caregivers.

### 2.4. Measures

The Preschool Language Scale–5th Edition (PLS–5). The Preschool Language Scale–5th Edition (PLS–5) [31] is a standalone, norm-referenced developmental language assessment designed to evaluate auditory comprehension and expressive language in children from birth through 7 years, 11 months. This English-language measure yields standard scores, percentile ranks, and age-equivalent scores for auditory comprehension, expressive language, and total language ability. The PLS–5 was standardized on a sample of more than 1400 children and demonstrates adequate diagnostic accuracy, with reported sensitivity of 0.83 and specificity of 0.80 for identifying language delays [34]. The measure has also demonstrated strong psychometric properties, including good internal consistency among low-income English- and Spanish-speaking children [35].

Peabody Developmental Motor Scales, Second Edition (PDMS-2). Motor development was assessed using the PDMS-2 [36], a standardized measure for children from birth to 71 months. The full assessment, including both fine and gross motor subtests, was administered to evaluate overall motor skills and guide eligibility, individualized intervention planning, and progress monitoring. The PDMS-2 demonstrates strong psychometric properties, including high internal consistency (ICC 0.84–0.99) [37], test–retest reliability (0.75–0.99 for Gross Motor; 0.71–0.99 for Fine Motor), and interrater reliability as well as established content and construct validity [38].

Devereux Early Childhood Assessment (DECA). Behavior functioning was assessed using the DECA [39], a caregiver-completed rating scale for children birth to five years. The DECA evaluates protective factors (Attachment, Initiative, Self-Regulation) and Behavior Concerns, producing a Total Protective Factors score. Scores guided eligibility, individualized behavioral interventions, and tracking of behavioral progress. Psychometric properties are well-established. The DECA demonstrates strong psychometric properties, including adequate to excellent internal consistency (0.80–0.95), test–retest reliability (0.78–0.95), and evidence of construct and criterion-related validity (*p* < 0.01) [40].

Brigance Inventory of Early Development (Brigance). General development was assessed using the Brigance [41], a criterion-referenced measure for children from birth through early childhood. The Brigance evaluates multiple domains, including language, motor, cognitive, social-emotional, and adaptive. Scores informed eligibility, individualized developmental goals, and ongoing progress monitoring. The Brigance demonstrates strong utility for early intervention due to its developmental sensitivity, flexibility of administration, and alignment with functional skill development. Psychometric properties are well-established. The Brigance demonstrates strong psychometric properties, including excellent internal consistency (0.94–0.97), test–retest reliability (0.98–0.99), inter-rater reliability (0.98–0.99) and evidence of construct and criterion-related validity [41].

### 2.5. Demographic Indicators

Demographic information was collected during an initial enrollment phone call conducted by program coordinators. Calls were completed in English, Spanish, or Creole based on caregiver preference.

Child Indicators. Caregivers reported their child’s date of birth (used to calculate age in months at program entry), gender (coded 0 = male, 1 = female), race (Asian, Black or African American, Pacific Islander, White, Biracial or Multiracial, Other), and ethnicity (Latine, Haitian, Both, Neither). Gestation at birth was recorded and coded to indicate prematurity (0 = ≥37 weeks; 1 = <37 weeks). Caregivers also reported their child’s health insurance status (private, government-assisted, uninsured).

Family Indicators. Caregivers reported their date of birth (used to calculate age at the child’s birth), country of birth (recoded as 0 = outside the U.S., 1 = within the U.S.), and languages spoken, which was used to indicate comfort communicating in English (0 = not comfortable; 1 = comfortable). Relationship status was recorded and recoded (0 = married or common union; 1 = single, divorced, separated, or widowed). Caregivers also reported their highest level of education, household composition (number of adults and children), and annual household income using program-defined brackets ($0–$70,000; $70,001–$110,000; ≥$110,001).

### 2.6. Procedures

Following referral, children completed an initial screening to determine eligibility and assign an appropriate intervention service type based on the referral packet (speech-language, occupational, behavioral, or general developmental intervention). During intake, caregivers completed demographic questionnaires and brief surveys, and program coordinators collaborated with families to identify priorities and coordinate services with a community-based provider. Standardized baseline assessments were administered by trained clinicians prior to the initiation of intervention to establish eligibility and develop individualized goals that guide each child’s intervention plan. All children who met eligibility criteria were enrolled consecutively during the 2024–2025 fiscal year.

Children who met eligibility criteria and whose caregivers agreed to participate received short-term intervention consisting of 8–20 sessions delivered over approximately 4–6 months. Services were provided 1–3 times per week, with sessions lasting 30–60 min, and occurred in community settings including childcare centers, homes, provider clinics, via telehealth, or other community setting (e.g., local library or playground). Delivery modality was not examined analytically, as the study focused on overall intervention implementation and outcomes rather than differences across service formats. Although telehealth was included as a delivery model, it accounted for less than 7% of cases and was therefore not analyzed separately due to the small sample size. A family care coordinator maintained regular contact with caregivers throughout the intervention period to monitor progress, address concerns, and support family engagement. The number of sessions provided was determined based on clinical judgment depending on rate of progress toward individualized goals and provider recommendation.

Post-intervention assessments were administered at program discharge using the same standardized measures selected at baseline to evaluate developmental change. All assessments and intervention services were delivered by licensed or supervised professionals consistent with the child’s assigned service type. Assessors were aware of children’s participation in intervention, as assessments were conducted as part of routine program implementation; however, standardized administration and scoring procedures were followed to support consistency. Although service duration varied between 4–6 months depending on attendance and session frequency, assessment followed a consistent pre-post structure across all participants. Pre-intervention assessments were completed following enrollment and prior to initiation of services, and post-intervention assessments were conducted at the conclusion of each child’s individualized service plan rather than at a fixed calendar interval. Standardized administration procedures were followed for all measures. No systematic procedural variations occurred across service types.

To promote high quality intervention services and promote consistency across providers and service types, fidelity check reviews are conducted at least twice annually for all interventionists delivering intervention services to children enrolled in the ED Program. Fidelity checks consisted of a direct observation of a live session or review of a recorded session. Fidelity checks were evaluated by the Program Manager using a fidelity checklist which included session structure, coaching strategies, adherence to service type framework, and developmentally appropriate materials and strategies. Fidelity reviews were conducted as part of routine quality assurance procedures. Inter-reliability was not assessed as each fidelity check was conducted by a single reviewer and not scored. Additionally, checks were not included as analytic variables.

### 2.7. Statistical Analyses

Preliminary analyses were conducted to examine descriptive statistics of study variables, including frequencies, means, standard deviations, and ranges. Next, significance of associations between study variables were examined using Phi and Cramer’s V values for relationships between two categorical variables and point biserial correlations for relationships between one categorial and one continuous variable. Then, Chi-Square Tests for Independence were used to explore relationships between categorical indicators and improvement (with Yates Continuity Correction to compensate for the overestimate of the chi-square value in 2 × 2 comparisons and Fisher’s Exact Probability Test when expected cell counts violated assumptions), and logistic regressions were performed to assess the impact of continuous predictors on the likelihood that children would exhibit significant improvement. Given the number of bivariate tests conducted, analyses examining associations between improvement and program, child, and family indicators should be considered exploratory. Significant improvement was defined as either (1) movement from one performance classification category to at least the next classification (e.g., below average to average) in at least one developmental domain, or (2) an increase of at least 5 standard score points above baseline in at least one domain that initially indicated a mild developmental delay (standard score ≤ 84), with maintenance of scores at or above 85 in all other domains, indicating the absence of mild developmental delays. Criteria for improvement were established by the program developers and funders prior to data collection and were applied consistently across participants. This definition, established in collaboration with the grant funders and developers, was intended to reflect clinically meaningful change and to align with programmatic goals focused on functional developmental progress. However, we acknowledge that dichotomizing improvement may reduce sensitivity to gradations of change and does not capture the full distribution of score variability. For example, a child may have demonstrated incremental gains that were not reflected in the dichotomous indicator. Inverse criteria to categorize children as demonstrating worsening functioning were not applied. Children who did not meet criteria for improvement were grouped together and included those whose scores maintained or worsened. Factors may have included need for longer-term intervention or lack of caregiver engagement. Sensitivity analyses using more stringent criteria for improvement (an increase of at least 10 standard score points above baseline in domains that indicated mild developmental delays [standard score ≤ 84], with maintenance of scores at or above 85 in all other domains, indicating the absence of mild developmental delays) were conducted to examine the robustness of findings.

## 3. Results

### 3.1. Power Analyses

A priori power analyses were conducted using G*Power 3.1 to determine the sample size required to detect a medium effect size (*w* = 0.30) with α = 0.05 and power set to 0.80. The results indicated a minimum required sample size of 88 for a 2 × 2 chi-square test (*df* = 1), 108 for 2 × 3 chi-square test (*df* = 2), and 122 for 2 × 4 chi-square test (*df* = 3). In addition, an a priori power analysis was performed using G*Power 3.1 to determine the sample size for logistic regression with one normal predictor. With α = 0.05, power set to 0.80, and a baseline probability of 0.50 for improvement, the results indicated that a total sample of 208 was required to detect an odds ratio of 1.5 or larger.

### 3.2. Program Indicators

Most children completed the ED Program for speech-language delays (*n* = 234; 68%), followed by occupational (*n* = 47; 14%), general developmental (*n* = 42; 12%), and behavior (*n* = 19; 6%). Children received between 8 and 20 sessions during the program (M = 14; Md = 12). Overall, 85% of children who completed the program demonstrated significant improvement. The remaining 15% of children either demonstrated worsening functioning or remained stable (i.e., did not meet criteria for improvement).

### 3.3. Child and Family Indicators

Children ranged from 1 to 71 months (M = 28, SD = 13.96) and were predominantly male (68%). The sample was racially and ethnically diverse (82% White, 13% Black, 4% Biracial or Multiracial; 74% Latine; 6% Haitian; 0.3% Both; 20% Neither). About 15% were born prematurely, 32% had government-assisted insurance, and 15% were uninsured. Caregivers’ average age was 32 years old (SD = 5.61), 55% were born outside the U.S., 21% reported limited English proficiency, and 30% were single caregivers. The majority of households reported incomes <$70,000 (71%). See Table 2 and Table 3.

### 3.4. Associations Between Improvement and Program, Child, and Family Indicators

#### 3.4.1. Improvement by Program Indicators

Improvement did not appear to differ by service type, χ^2^ (1, *n* = 342) = 1.06, *p* = 0.79, Cramer’s V = 0.06. It was high across all services: language (86%), occupational (81%), general developmental (83%), and behavior (84%).

The logistic regression model examining number of sessions was not statistically significant and explained minimal variance in improvement, χ^2^(5) = 8.60, *p* = 0.13. The model explained only 1% (Nagelkerke R^2^) of the variance in improvement and correctly classified 75% of cases. Further, number of sessions did not emerge as a significant predictor of improvement, Exp(B) = 0.06, *p* = 0.21, 95% CI [0.97–1.16].

#### 3.4.2. Improvement by Child Indicators

**Figure 1:**
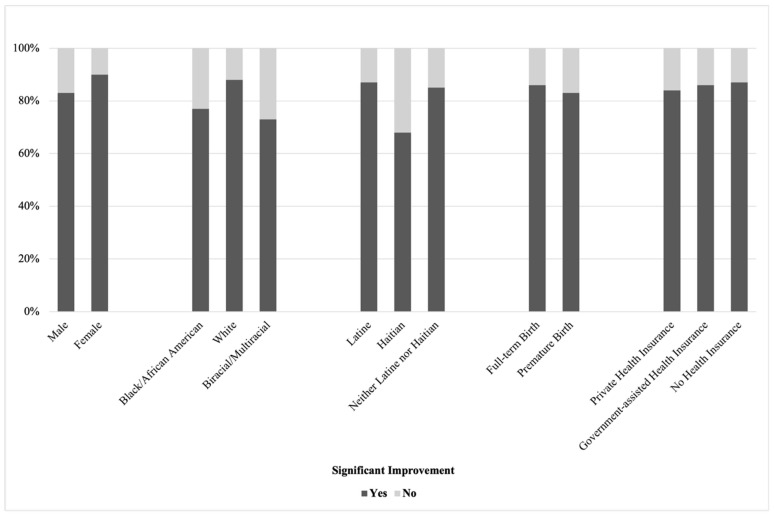
Improvement by Categorical Child Indicators. Note. The first set of bars represents child gender, the second set represents child race, the third set represents child ethnicity, the fourth set represents child length of gestation, and the fifth set represents child health insurance status. Improvement depicted for racial and ethnic categories comprising >1% of the sample.

**Figure 2:**
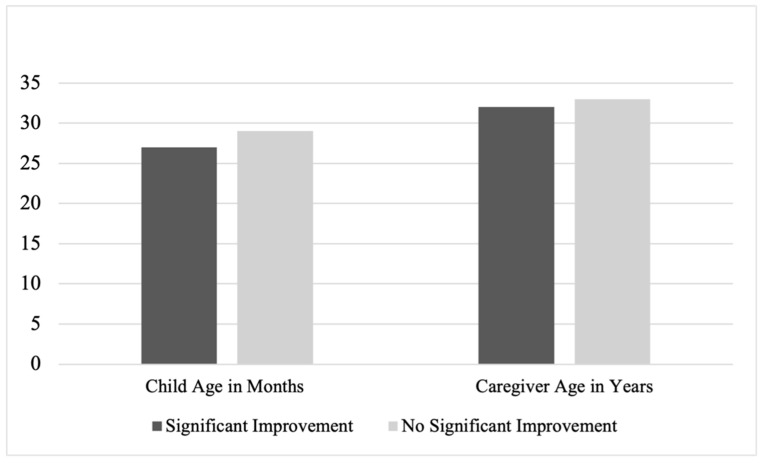
Improvement by Child and Caregiver Age. Note. The vertical axis represents child age in months and caregiver age in years.

A significant association emerged between improvement and child ethnicity, χ^2^ (1, *n* = 342) = 10.33, *p* = 0.02, Cramer’s V = 0.17. In this sample, Haitian children were less likely to demonstrate significant improvement (*n* = 13) compared to Latine children and children who were neither Latine nor Haitian (*n* = 278), χ^2^ (1, *n* = 342) = 6.76, Fisher’s Exact Test *p* = 0.02, *phi* = −0.14. Specifically, 65% of Haitian children demonstrated significant improvement, whereas 86% of non-Haitian children demonstrated significant improvement. No significant associations were found between improvement and child age, gender, race, prematurity, or insurance status (all *p* > 0.08). See Figure 1 and Figure 2.

#### 3.4.3. Improvement by Family Indicators

Logistic regression models examining caregiver age at the child’s birth, χ^2^(8) = 3.81, *p* = 0.87, and household size, χ^2^(3) = 3.90, *p* = 0.27, were not statistically significant and explained minimal variance in improvement (<1% and 1%, respectively), despite correctly classifying 86% and 85% of cases, respectively. Neither caregiver age, Exp(B) = 1.00, *p* = 0.95, 95% CI [0.95–1.05], nor household size, Exp(B) = 0.81, *p* = 0.10, 95% CI [0.64–1.04], emerged as significant predictors.

**Figure 3:**
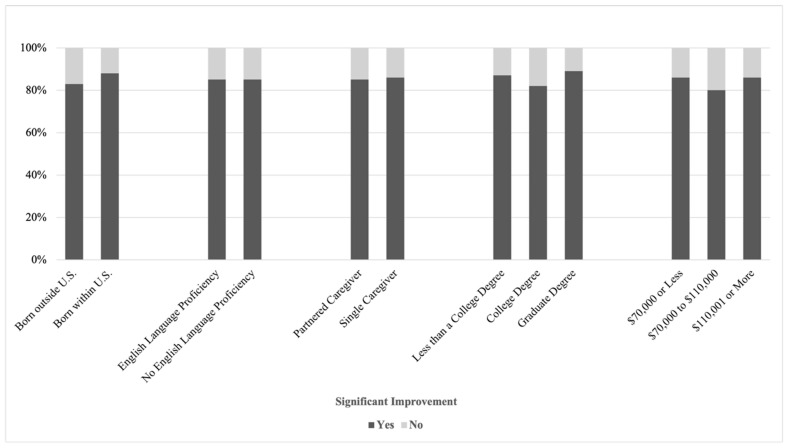
Improvement by Categorical Family Indicators. Note. The first set of bars represents caregiver country of birth, the second set represents caregiver English language proficiency, the third set represents caregiver relationship status, the fourth set represents caregiver education level, and the fifth set represents household income.

No significant associations emerged between improvement and caregiver country of birth, English language proficiency, relationship status, education level, or household income. Rates of significant improvement were comparable across all family demographic groups, suggesting that program success was consistent regardless of caregiver or household characteristics (all *p* > 0.23). See Figure 2 and Figure 3.

### 3.5. Sensitivity Analyses

Sensitivity analyses using more stringent criteria for improvement (an increase of at least 10 standard score points above baseline in domains that indicated mild developmental delays, with maintenance of scores in all other domains, indicating the absence of mild developmental delays) generally yielded results consistent with the primary analyses. Improvement by program indicators (service type and number of sessions; all *p* > 0.47) and family indicators (caregiver age, country of birth, English language proficiency, relationship status, and education level; household size and income) remained non-significant (all *p* > 0.08). Similarly, improvement by most child indicators remained non-significant (child gender, race, prematurity, and insurance status; all *p* > 0.07). Improvement by child ethnicity was no longer statistically significant when using more stringent improvement criteria, χ^2^ (1, *n* = 342) = 4.20, *p* = 0.24, Cramer’s V = 0.11. Lastly, a significant association appeared to emerge between improvement and child age, χ^2^(8) = 18.54, *p* = 0.02. However, the model only classified 63% of cases correctly and only explained 8% of the variance in improvement. The mean age of children who showed improvement when using more stringent criteria was 25 months, whereas the mean age of children who did not show improvement was 32 months. In comparison, the primary analyses using the original indicator of improvement revealed a mean age of 27 months for children who showed improvement and a mean age of 31 months for children who did not show improvement.

## 4. Discussion

The current study examined developmental outcomes associated with participation in the ED Program, a short-term, community-based early intervention model designed to serve young children with mild developmental delays who do not qualify for federally funded Part C or Part B services. Overall, findings indicate high rates of observed improvement, with the majority of children (85%) demonstrating significant improvement from pre- to post-intervention across service types. Although changes may also be due to developmental maturation, increased caregiver awareness, or other contextual influences, the RTI-informed framework guiding the intervention emphasizes systematic progress monitoring and responsiveness to treatment, supporting the understanding that observed gains reflect meaningful developmental change beyond expected maturation. These findings align with prior work showing that brief, targeted early intervention can meaningfully support developmental progress during a highly malleable period of early childhood. These findings also reflect existing literature demonstrating the benefits of early intervention during critical periods of high neuroplasticity for children with mild delays following brief, targeted intervention [1]. Prevention research similarly suggests that participation in early intervention may be associated with developmental progress prior to school entry [17,42].

Sensitivity analyses using a more stringent criteria for improvement (+10 standard score points) generally supported the primary findings. Notably, child age emerged as a potential predictor of improvement under the stricter criteria, with younger children more likely to demonstrate improvement. This finding is consistent with the literature indicating that earlier intervention is associated with improved developmental outcomes [43].

### 4.1. Program Improvement Across Service Types

Consistent with our hypotheses, children appeared to demonstrate high rates of improvement regardless of service type, including speech-language, occupational, general developmental, and behavioral interventions. Importantly, no significant differences in improvement emerged across service types, suggesting short-term intervention may be broadly effective for addressing mild delays across multiple service types. This finding aligns with prior research demonstrating that early, focused intervention can produce meaningful developmental gains even when delivered over relatively brief periods [17,42].

The absence of service-type difference also highlights the potential value of individualized, needs-based intervention planning within community-based programs. Rather than relying on diagnosis-driven eligibility or intensity requirements, the ED Program prioritizes functional need and early risk indicators, allowing children to receive timely support before delays escalate. These findings contribute to a growing body of literature suggesting prevention-oriented, community-based programs can address service gaps for children who do not qualify for intensive federally funded services [14,15].

Similar outcomes across service types may reflect shared components of the ED Program rather than differences between specific interventions. Across all services, intervention included individualized goal setting, caregiver involvement and coaching, and delivery in the child’s natural environment. The ED Program was designed using an RTI-informed framework, emphasizing individualized goal setting, continuous progress monitoring, and responsiveness to child performance and family functioning. These common elements may promote developmental progress by helping caregivers support their child’s learning, increasing opportunities for skill practice in daily routines, and strengthening foundational skills that affect multiple areas of development. These shared components align with core RTI principles and suggest that improvements observed across service types may reflect the success of an individualized, responsive, and prevention-oriented model rather than discipline-specific strategies alone. Although session dosage varied across participants, ranging from 8–20 sessions, dosage was not a significant predictor of improvement outcomes. This is consistent with prior early intervention research showing that more sessions do not always lead to better outcomes [44,45]. The ED Program was developed as a brief, targeted intervention for young children with mild delays, and it is possible that many children responded within the short-term model. Another possibility is that the emphasis on caregiver coaching and delivery in natural environments may have supported continued skill development beyond service delivery.

### 4.2. Equity and Developmental Outcomes

A main point of this study was to examine whether the ED Program supported young children across diverse demographic and socioeconomic backgrounds. Results largely support this aim. Improvement was not significantly associated with child age, gender, race, prematurity, insurance status or the majority of family-level indicators, including caregiver education, income, relationship status, household size, country of birth, or English language proficiency. These findings suggest that observed pre-post improvements were comparable across many child and family characteristics in this sample.

This pattern is noteworthy given that many families in the sample reported low household income, and limited insurance coverage, both of which have been consistently linked to reduced access to early intervention and poorer developmental outcomes [11,12]. Additionally, by offering low- or no-cost services, providing linguistically responsive care, and embedding intervention within community and naturalistic settings, the ED Program may help to reduce barriers that disproportionately affect historically marginalized families. This is consistent with literature describing the potential advantages of flexible, community-embedded models for reaching families who are less likely to receive or sustain services in more restrictive systems [3,10]. It is important to note that subgroup sample sizes may have limited statistical power to detect small-to-moderate effects, therefore, the absence of significant differences should be interpreted cautiously.

### 4.3. Improvement Outcomes by Ethnicity

An exception related to equitable outcomes was an observed difference in improvement rates among Haitian children compared to Latine children and children who were neither Latine nor Haitian in the primary analyses. However, this association was not maintained in sensitivity analyses when using a more stringent criteria for improvement, suggesting this finding may not be robust and warrants further investigation. Additionally, Haitian families represented a small proportion of the sample, limiting statistical power and the reliability of subgroup comparisons. Therefore, these should not be interpreted as definitive evidence of differential effectiveness. Caregiver English proficiency was not associated with improvement, suggesting that language fluency alone does not explain this observed difference. Given the small number of Haitian children in the sample, the present study was not designed or powered to examine cultural, linguistic, or systemic factors within this subgroup. These results highlight the importance of continued monitoring of subgroup outcomes as part of ongoing program evaluation.

### 4.4. Implications

Findings from the current study have several important implications for early childhood service delivery and intervention policy. Results indicate that short-term, community-based early intervention programs may provide meaningful developmental support for children with mild developmental delays who do not qualify for federally funded services. This is particularly significant given that children with mild or emerging delays are often overlooked despite evidence that early support can positively influence developmental trajectories and lessen the need for more intensive services later [9]. By addressing gaps in access to early intervention, community-based models may represent a promising approach for supporting early development and strengthening existing service systems. Although these findings provide preliminary evidence for feasibility and potential benefit, they reflect program-level evaluation rather than definitive evidence for large-scale policy reform. Broader policy or funding recommendations would require controlled comparative studies, longitudinal follow-up, and cost-effectiveness analyses to establish scalability and sustained impact.

The ED Program represents an adaptable model that reinforces existing early intervention systems by filling a critical service gap. Rather than replacing IDEA Part C or Part B services, community-based programs such as ED may serve as an early, preventive intervention, supporting school readiness and potentially minimizing future eligibility for IDEA services. These findings contribute to the growing, but still limited, research on community-based programs for children with mild developmental delays. It also highlights the need for sustainable funding mechanisms to support such initiatives.

## 5. Limitations

While this study provides important insights, several limitations should be considered when interpreting the findings of this study. First, improvement was determined using program-specific criteria based on standardized pre- and post-test assessments. Although these measures are widely used and psychometrically sound, reliance on program-defined thresholds may limit comparability with other studies. In addition, the use of a binary indicator of improvement possibly obscured variability and limits the interpretability of study findings. Examining continuous indicators of improvement in future work would provide greater nuance to our understanding of program success. Second, the absence of a control or comparison group precludes causal inference. Without a non-intervention group, it is not possible to rule out maturation or regression to the mean as contributors to observed improvements. However, the magnitude and consistency of gains across service types provide preliminary support for continued evaluation of the program model. Additionally, the structure of the present sample did not readily allow for clustering by service provider or service type, as providers delivered varying combinations of services across participants. Future studies may benefit from examining clustering by service provider and service type to further evaluate potential dependence among observations. Third, generalizability may be limited to similar urban, community-based contexts serving low-income and diverse populations. Replication in other geographic regions and service systems is needed to establish broader applicability. The relatively small proportion of Haitian children limits statistical power and may contribute to instability in subgroup comparisons. Moreover, the numerous bivariate statistical tests conducted increases the overall likelihood of Type I error, meaning some significant findings may reflect chance rather than true effects. Results should therefore be interpreted cautiously and replicated in future studies. Lastly, the logistic regression models explained minimal variance. Future research should explore alternative predictors of improvement, such as caregiver engagement, baseline functioning, intervention intensity, and fidelity, to better understand variability in treatment response.

## 6. Future Directions

Future research incorporating comparison or waitlist control groups would strengthen causal inference and allow clearer attribution of outcomes to program participation. Although telehealth was included as a service delivery approach, outcomes were not examined by service format (e.g., telehealth vs. in-person), which may have implications for effectiveness and equity. Longitudinal follow-up through kindergarten entry could clarify the durability of observed gains, and future work may also examine dose–response relationships and include independent fidelity ratings with interrater reliability assessment to strengthen evaluation of implementation quality.

## 7. Conclusions

Results provide preliminary evidence that participation in the ED Program was associated with developmental improvement among young children with mild developmental delays who are often excluded from federally funded early intervention services. High rates of improvement across service types and largely equitable outcomes across demographic and socioeconomic groups highlight the potential of short-term, community-based intervention models to support early development during a critical period of neuroplasticity.

Although situated within a specific local context, these findings have broader conceptual relevance. Early intervention systems vary substantially across countries in terms of eligibility criteria, funding, and service intensity. Accordingly, findings from this study should not be interpreted as directly generalizable across national contexts but may instead offer conceptual insight for systems addressing similar services gaps. Many early childhood systems worldwide face challenges related to service eligibility, delayed access to care, and unmet developmental needs among children with mild or emerging delays. Results from this study suggest that brief, community-embedded early intervention programs may serve as a complementary strategy within existing early childhood health and education systems.

By addressing gaps in access to early intervention, community-based programs such as ED may contribute to improved school readiness and reduced developmental inequities. These findings contribute to ongoing discussions regarding the potential role of preventive, short-term early intervention models within national and regional early childhood systems.

## Figures and Tables

**Table 1:** Child and Family Demographic Information.

Child Indicators						
Race	Asian	Black or African American	Pacific Islander	White	Biracial or Multiracial	Other
	<1%	13%	<1%	82%	4%	<1%
Ethnicity	Latine	Haitian	Both	Neither		
	74%	6%	<1%	20%		
Prematurity	≥37 weeks	<37 weeks				
	85%	15%				
Health Insurance	Private	Government-assisted	Uninsured			
	53%	32%	15%			
Family Indicators						
Country of Birth	Outside the U.S.	Within the U.S.				
	55%	45%				
English Proficiency	No	Yes				
	21%	78%				
Relationship Status	Partnered	Single				
	70%	30%				
Education Level	<Two-year Degree	Two- or Four-year Degree	Graduate Degree			
	25%	44%	27%			
Income	$0–$70,000	$70,001–$110,000	≥$110,001			
	71%	10%	19%			

**Table 2:** Means, Standard Deviations, and Associations between Improvement and Child Indicators.

Variable	*M*	SD	1	2	3	4	5	6
1. Improvement	0.85	0.36						
Child Indicators								
2. Age in Months	27.80	13.96	−0.06					
3. Gender	0.32	0.47	0.10	−0.08				
4. Race			0.12	0.01	0.04			
5. Ethnicity			0.17 *	0.001	0.05	0.54 **		
6. Prematurity	0.15	0.36	−0.03	−0.23 **	0.11 *	0.06	0.05	
7. Health Insurance			0.03	−0.22 **	0.03	0.14 *	0.13	0.10

Note. M *=* Mean; SD = Standard Deviation. Improvement: 0 = No improvement, 1 = Improvement. Gender: 0 = Male, 1 = Female. Race: Asian, Black or African American, Pacific Islander, White, Biracial or Multiracial, Other. Ethnicity: Latine, Haitian, Neither, or Both. Prematurity: 0 ≥ 37 weeks’ gestation, 1 < 37 weeks’ gestation. Health Insurance: Private health insurance, Government-assisted health insurance, No health insurance. Associations between two categorical variables represented by Phi or Cramer’s V values and between one categorial and continuous variable represented by point biserial correlations. * *p* < 0.05; ** *p* < 0.01.

**Table 3:** Means, Standard Deviations, and Associations between Improvement and Family Indicators.

Variable	*M*	SD	1	2	3	4	5	6	7
1. Improvement	0.85	0.36							
Caregiver									
2. Age in Years	32.47	5.61	−0.003						
3. Country of Birth	0.45	0.50	0.07	−0.04					
4. English Proficiency	0.79	0.41	0.01	−0.001	0.44 **				
5. Relationship Status	0.30	0.46	0.02	−0.11 *	0.14 *	0.04			
6. Education Level			<0.001	0.15 **	−0.01	0.14*	−0.36 **		
Household									
7. Size	3.70	1.11	−0.09	0.20 **	0.06	0.01	−0.28 **	0.05	
8. Income			0.05	0.11	0.10	0.24**	0.26 **	0.39 **	0.04

Note. M = Mean; SD = Standard Deviation. Improvement: 0 = No improvement, 1 = Improvement. Country of Birth: 0 = Outside the U.S., 1 = Within the U.S. English Proficiency: 0 = Not comfortable communicating in English, 1 = Comfortable communicating in English. Relationship Status: 0 = Partnered caregiver, 1 = Single caregiver. Education Level: Less than high school diploma, High school diploma/Equivalent, Some college, Associate’s degree, Bachelor’s degree, Graduate degree. Size: Total number of adults and children in the household. Income: $0 to $70,000, $70,001 to $110,000, or $110,001 or more. Associations between two categorical variables represented by Phi or Cramer’s V values and between one categorial and continuous variable represented by point biserial correlations. * *p* < 0.05; ** *p* < 0.01.

## Data Availability

The data presented in this study are openly available in ED Outcomes Data (Box Link) and was accessed on 2 February 2026.

## Abbreviations

The following abbreviations are used in this manuscript:

ED	Early Discovery
IDEA	Individuals with Disabilities Education Act
PLS–5	Preschool Language Scale–5th Edition
PDMS-2	Peabody Developmental Motor Scales, Second Edition
ESDM	Early Start Denver Model
DECA	Devereux Early Childhood Assessment
PCIT	Parent-Child Interaction Therapy
PMT	Parent Management Training
